# Case report: A rare case of pyruvate kinase deficiency and Crigler-Najjar syndrome type II with a novel pathogenic variant of *PKLR* and *UGT1A1* mutation

**DOI:** 10.3389/fgene.2023.1229271

**Published:** 2023-08-21

**Authors:** Huan Wu, Long Wu, Quan Zhang, Bao-fang Zhang

**Affiliations:** ^1^ Department of Infectious Diseases, The Affiliated Hospital of Guizhou Medical University, Guiyang, Guizhou, China; ^2^ Department of Anus and Intestinal Surgery, The Affiliated Hospital of Guizhou Medical University, Guiyang, Guizhou, China

**Keywords:** pyruvate kinase deficiency, Crigler-Najjar syndrome, next-generation sequencing, PKLR, UGT1A1, case report

## Abstract

Pyruvate Kinase Deficiency (PKD) and Crigler-Najjar syndrome are rare autosomal recessive liver diseases. PKD is caused by homozygous or compound heterozygous mutations in the *PKLR* gene, leading to non-spherocytic hereditary hemolytic anemia. On the other hand, Crigler-Najjar syndrome (CNS-II) is characterized by the loss or reduced activity of UDP-glucuronosyltransferase, resulting in elevated levels of unconjugated bilirubin, which is the primary cause of disease manifestation. To date, there have been no reported cases of patients with both conditions. In this case report, we present the unique clinical course of a 15-year-old Chinese patient with both PKD and CNS-II. The patient was admitted for evaluation of hyperbilirubinemia and exhibited yellowish skin color, icteric sclera, and splenomegaly upon physical examination. Extensive laboratory examinations ruled out viral, hemolytic, autoimmune, and inborn or acquired metabolic etiologies of liver injury. Histopathological findings indicated benign recurrent intrahepatic cholestasis (BRIC) and hemosiderosis. Surprisingly, targeted next-generation sequencing (NGS) of the patient’s blood did not reveal any mutation sites associated with BRIC. Instead, it identified a novel homozygous pathogenic variant of the *PKLR* gene [c.1276C>T (p.Arg426Trp)] and a rare heterozygous variant of *UGT1A1* gene [c.-55_-54insAT, c.1091C>T (p.Pro364Leu)]. These findings strongly suggest a diagnosis of PKD and CNS-II in the patient. Treatment with 500 mg/day of ursodeoxycholic acid proved to be effective, rapidly reducing the patient’s total bilirubin levels and shortening the symptomatic period. This case highlights the importance of genetic diagnosis in accurately identifying the underlying cause of hyperbilirubinemia, especially in patients with rare hereditary diseases. Furthermore, NGS can provide valuable insights into the genotype-phenotype correlation of PKD and CNS-II.

## Introduction

Hyperbilirubinemia is a medical condition characterized by elevated levels of bilirubin in the bloodstream, exceeding the reference range established by laboratory standards. It is primarily caused by disorders affecting the metabolism of bilirubin. Bilirubin metabolism involves the uptake of bilirubin from circulation, its storage within cells, conjugation with glucuronic acid, and subsequent excretion into bile. Any abnormalities in these processes can result in hyperbilirubinemia (Bhandari et al., 2023).

Pathogenic variants in the *PKLR* and *UGT1A1* genes are responsible for causing Pyruvate Kinase Deficiency (PKD) and Crigler-Najjar syndrome, respectively. PKD is an autosomal recessive genetic disorder characterized by various clinical manifestations, including intrauterine growth retardation, jaundice, cholelithiasis, cholecystitis, splenomegaly, and chronic hemolytic anemia. Patients with homozygous nonsense mutations often experience the most severe form of the disease. Crigler-Najjar syndrome is a rare autosomal recessive disorder characterized by non-hemolytic unconjugated hyperbilirubinemia, resulting from a deficiency in UDP-glucuronosyltransferase. Unfortunately, both PKD and CNS-II can exhibit symptoms similar to other diseases, such as dull pain in the liver area, generalized weakness, fatigue, and loss of appetite, leading to potential misdiagnosis. Furthermore, the erythrocyte morphology in PKD is typically normal, and even the detection of pyruvate kinase activity may appear normal, making the diagnosis of PKD and CNS-II challenging. The definitive diagnosis relies on sequencing the *PKLR* and *UGT1A1* genes through conventional or targeted next-generation sequencing methods, which involve analyzing genes associated with enzymopathies, membranopathies, hemoglobinopathies, and bone marrow failure disorders. It is worth noting that despite being a rare hereditary disease, routine laboratory examinations and physical examinations often lack the necessary diagnostic tools to detect this syndrome. This scarcity of experience in clinical practice poses challenges for non-specialist physicians when encountering these conditions.

We report the case of a patient exhibiting jaundice, characterized by significantly elevated bilirubin levels. Following the exclusion of common diseases, a liver tissue biopsy was performed. Histopathological examination revealed benign recurrent intrahepatic cholestasis (BRIC) and hemosiderosis. However, through next-generation sequencing, we confirmed that the patient had both Pyruvate Kinase Deficiency (PKD) and Crigler-Najjar Syndrome type II (CNS-II). The sequencing results indicated a rare homozygous missense mutation in the *PKLR* gene and a rare heterozygous variant in the *UGT1A1* gene. Our aim with this report is to emphasize the importance of genetic testing in patients presenting with unexplained jaundice. The identification of new *UGT1A1* mutations associated with CNS-II and novel *PKLR* mutations linked to PKD expands our understanding of the mutational landscape associated with these rare Mendelian disorders. By highlighting the role of genetic testing, we contribute to the broader knowledge of these conditions and their underlying genetic mechanisms.

## Case presentation chief complaints

A 15-year-old Chinese boy had been experiencing persistent “hyperbilirubinemia” for a period of 3 years, seeking medical attention at various local hospitals. Despite numerous visits, the underlying cause of the condition remained undiagnosed. The patient’s serological and laboratory results revealed elevated levels of total bilirubin (565.02 μmol/L), direct bilirubin (117.08 μmol/L), indirect bilirubin (447.94 μmol/L), alanine aminotransferase (349.8 U/L), aspartate aminotransferase (219.6 U/L), gamma-glutamyl transferase (253.3 U/L), alkaline phosphatase (165.0 U/L), and total bile acid (94.6 μmol/L). Notably, the patient did not exhibit any accompanying symptoms such as weakness, skin pruritus, nausea, or aversion to oily substances throughout the course of the disease. Consequently, the patient was referred to our department for further diagnostic evaluation.

## History of past illness, personal and family history

The patient, a student, has no previous history of drug use, blood transfusions, allergies, smoking, alcohol consumption, or any known inborn or acquired metabolic conditions. However, it is worth noting that the patient has a history of tattoos. The patient denied any family history of liver diseases. He is the child of a nonconsanguineous marriage and has one brother who does not have any hematological disorders.

## Physical examination

The patient exhibited visible yellow discoloration of the skin, mucous membranes, and sclera, indicating jaundice. There were no apparent signs of anemia. Upon abdominal examination, the abdomen was found to be soft, without the presence of varicose veins or any signs of exposure. Palpation of the liver under the ribs revealed it to be non-palpable, and percussion of the hepatic region did not elicit significant pain. However, the spleen was palpable approximately 3 cm below the left costal area, exhibiting a clear margin, medium texture, and no tenderness. No other abnormalities were detected during the physical examination.

## Laboratory and imaging examinations

**FIGURE 1:**
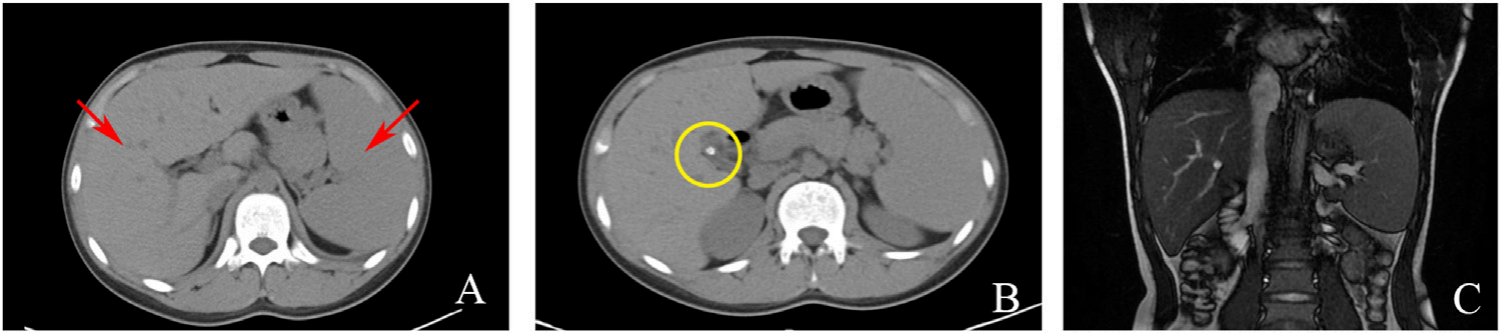
Imaging examinations: **(A)** CT scan showing liver and spleen enlargement (red arrow); **(B)** CT scan showing gallbladder neck stones (yellow circle); **(C)**. MRCP showing a normal intrahepatic and extrahepatic biliary tree and pancreatic ductal system.

Serological and laboratory investigations were conducted to rule out various conditions including viral hepatitis, autoimmune hepatitis, primary biliary cholangitis, Wilson disease, and liver damage associated with hyperthyroidism. Tests for herpes simplex virus, cytomegalovirus, and Epstein-Barr virus antibodies yielded negative results. Anemia-related tests, including those for hemolytic anemia, glucose-6-phosphate dehydrogenase deficiency (G-6-PD), paroxysmal nocturnal hemoglobinuria (PNH), and thalassemia genetic testing, were also negative. Blood routine analysis revealed mild anemia and reticulocytosis. Examination of the blood cell smear indicated slight variability in the size of red blood cells and the presence of polychromic red blood cells. Serum ferritin levels were elevated, while serum iron levels remained within the normal range (Table 1). Magnetic resonance cholangiopancreatography (MRCP) demonstrated normal intrahepatic and extrahepatic biliary tree structures, as well as a normal pancreatic ductal system (Figure 1). Additionally, a whole abdomen computed tomography (CT) scan revealed the presence of multiple gallbladder stones, increased liver parenchymal density, and an enlarged spleen (Figure 1).

**TABLE 1:** Laboratory data of patient with Pyruvate Kinase Deficiency on admission.

Method	Index	Result	Reference range
Blood routine	RBC (*10^12/L)	3.2	4.5–5.9
	HGB (g/L)	115	129–172
	MCV (fL)	107.2	80–100
	MCHC (g/L)	335.0	310–355
	MCH (pg)	35.9	25–34
	HCT (%)	34.3	39–51
	Ret (*10^9/L)	202.9	46.4–121.2
	Ret% (%)	6.34	0.9–2.22
Blood cell smear	Red blood cells are mildly unequal in size, polychromic red blood cells		
Liver function	ALT (U/L)	349.8	9–50
	AST (U/L)	219.6	15–40
	TBIL (μmol/L)	565.02	3.4–17.0
	DBIL (μmol/L)	117.08	≤8
	IBIL (μmol/L)	447.94	3.0–17.0
	GGT (U/L)	253.3	10–60
	ALP (U/L)	165.0	40–150
	TBA (μmol/L)	94.6	≤10

Note: RBC, red blood cell; HGB, hemoglobin; MCV, mean corpuscular volume; MCHC, mean corpuscular hemoglobin concentration; MCH, mean corpuscular hemoglobin; HCT, hematocrit; Ret, Reticulocyte; ALT, alanine transaminase; AST, aspartate aminotransferase; TBIL, total bilirubin; DBIL, direct bilirubin; IBIL, indirect bilirubin; ALP, alkaline phosphatase; GGT, gamma-glutamyl transferase; TBA, total bile acid.

## Liver pathological examinations

**FIGURE 2:**
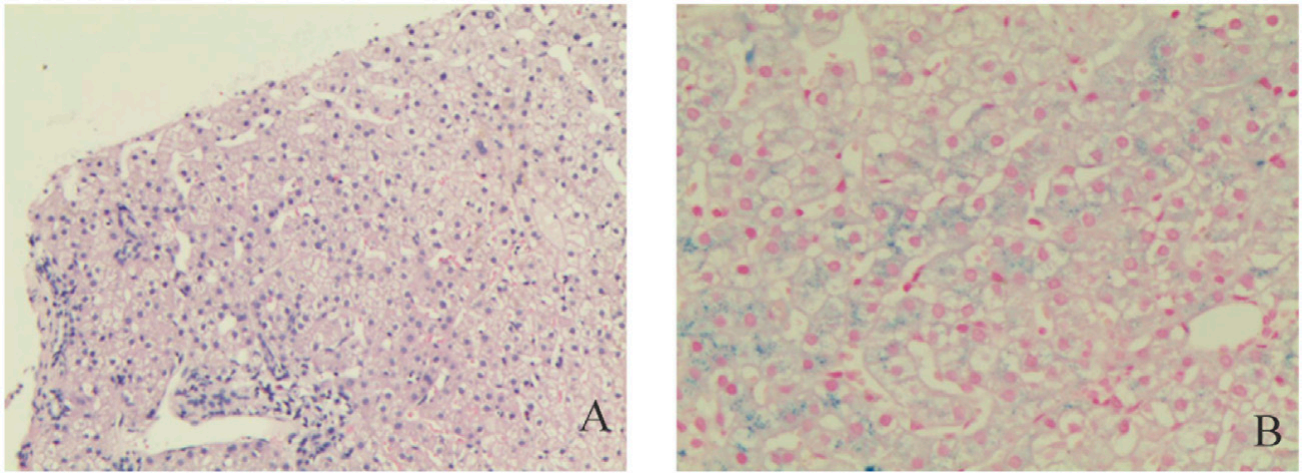
Pathological examinations. Hematoxylin and eosin staining liver cells with a large number of iron granules deposited in suspected Pyruvate Kinase Deficiency (PKD); **(A)** Original magnification ×10; **(B)** Original magnification ×200.

After excluding liver contraindications, an ultrasound-guided percutaneous liver biopsy was performed. The microscope showed marked diffuse watery degeneration of hepatocytes, cholestasis of hepatocytes around the central vein, bile thrombus in the capillary bile ducts, and a few mixed inflammatory cells in the hepatic sinusoids. No obvious abnormity was found in the portal area, fibrous tissue, bile ducts, and blood vessels between lobules (Figure 2). Further pathological assessment and immunohistochemistry studies revealed that CD10 was expressed at the canalicular membrane, CD68 was expressed at the canalicular membrane at the kupffer cells and portal macrophages, as well as a well-preserved intralobular bile duct arrangement (Table 1). Special staining of liver tissue showed positive for iron staining. However, no copper staining, PAS, reticular fiber staining, or collagen fiber staining (Masson) was found (Table 1). The final outcome considered BRIC and hemosiderosis.

## Further diagnostic workup

After obtaining informed consent, a genetic examination was performed to confirm the mutation sites of the *BRIC* gene and the clinical diagnosis.

## Molecular identification

**FIGURE 3:**
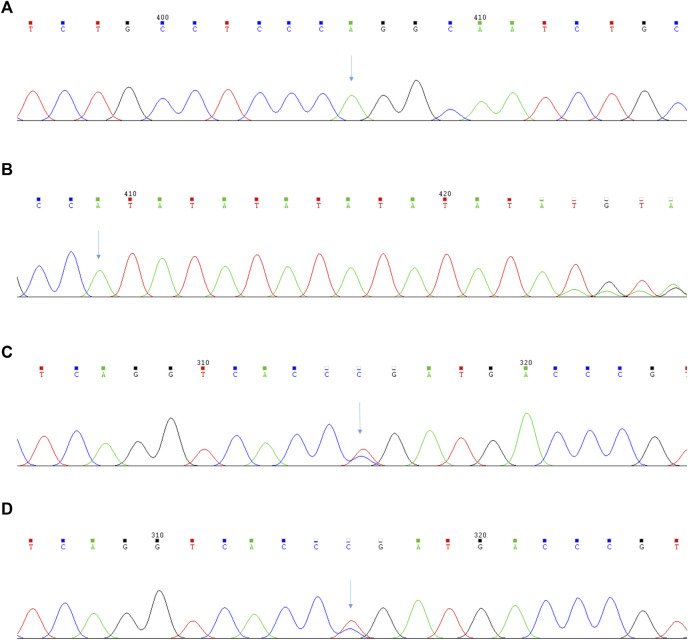
The results of the second-generation sequencing analysis of *PKLR* and *UGT1A1* gene in Pyruvate Kinase Deficiency patient. **(A)** The patient’s mutation sites of the *PKLR* gene: c.1276C>T (p.Arg426Trp); **(B)** The patient’s mutation sites of the *UGT1A1* gene:c.-55_-54insAT; **(C)** The patient’s mutation sites of the *UGT1A1* gene:c.1091C>T (p.Pro364Leu); **(D)** The patient’s brother’s mutation sites of the *UGT1A1* gene:c.1091C>T (p.Pro364Leu).

To our surprise, targeted NGS of blood did not identify the mutation sites of the *BRIC* gene but instead confirmed the presence of a novel homozygous pathogenic variant of c.1276C>T (p.Arg426Trp) in *PKLR* and a novel heterozygous pathogenic variant of c.-55_-54insAT, c.1091C > T (p.Pro364Leu) in *UGT1A1* (Figure 3). The mutation of the former was located on the exon of *PKLR* missense mutations; cytosine at position 1276 is replaced by thymine, which causes the conversion of arginine to tryptophan. The mutation of the latter was a novel heterozygous pathogenic variant and included base substitutions and base insertions. Base substitutions showed insertions of adenine and thymine at positions 54 and 55. Base insertions showed that cytosine at position 1091 is replaced by thymine, which causes the conversion of proline to leucine.

## Pathogenicity classification

1. We conducted a comprehensive search of various databases, including RefSeq, dbSNP, 1000Genome, gnomAD, ClinVar, ClinGen, OMIM, DECIPHER, NCBI Gene, and PubMed. The mutations identified in the patient have not been previously reported in population databases and are considered rare variants, suggesting moderate pathogenicity (PM2).
2. The identified mutations include homozygous missense mutation, heterozygous missense mutation, and heterozygous gene upstream mutation. Homozygous missense mutation affects the type and sequence of amino acids in the polypeptide chain, while heterozygous missense mutation reduces the promoter activity of the *UGT1A1* gene, thereby impacting its protein expression. Further analysis revealed that the mutation in the *UGT1A1* gene reduces enzyme activity by 64.4%. The scores obtained from REVEL and ClinPred software indicate a predicted harmful effect (>0.75 and >0.5, respectively). Additionally, protein function prediction using SIFT and Pyphen-2 software showed consistent results. Based on these findings, it is inferred that the carried mutations in the patient have an impact on the function of the resulting protein, providing moderate pathogenic evidence (PM2).
3. Another variant, NM_000298.6(*PKLR*):c.1277G>A (p.Arg426Gln), identified at a different amino acid position, is listed as a suspected pathogenic variant in the ClinVar database, indicating strong pathogenic evidence (PS1).
4. The patient is the child of a nonconsanguineous marriage and has no family history of the condition. The mutation was not detected in the next-generation sequencing data of the patient’s brother, confirming that these mutations are new in this patient. This observation provides moderate pathogenic evidence (PM6).

Based on the ACMG and AMP guidelines (Richards et al., 2015) for pathogenicity rating, considering the above variant evidence and evidence classification, these variations exhibit 3 moderate pathogenic evidences and 1 strong pathogenic evidence: 1PS+3PM, indicating a possible pathogenic variant.

## Final diagnosis

Thus, the patient was highly suspected to have PKD with CNS-II owing to these clinical, genetic, and pathological findings.

## Treatment

The patient was treated with ursodeoxycholic acid at a dose of 500 mg/day; its intermittent effect was obtained at the initial stage. Additionally, lipid-soluble vitamin (II) and water-soluble vitamins were used for nutritional support.

## Outcome and follow-up

The treatment showed positive results, as evidenced by significant improvements in the patient’s blood biochemical indices. The updated values are as follows: total bilirubin 189.6 μmol/L, direct bilirubin 31.7 μmol/L, indirect bilirubin 157.9 μmol/L, alanine aminotransferase 18.8 U/L, aspartate aminotransferase 17.5 U/L, gamma-glutamyl transferase 79.0 U/L, alkaline phosphatase 151.0 U/L, and total bile acid 3.25 μmol/L. The blood coagulation function showed no abnormalities, and the blood routine indicated elevated hemoglobin levels. The patient did not experience any discomfort. However, further long-term follow-up is necessary to monitor the patient’s prognosis. Unfortunately, the patient’s parents were unable to come to the hospital for examination, and thus, the mutation sites of *PKLR* and *UGT1A1* in the family could not be identified. Only the patient’s brother underwent NGS, which also did not identify the mutation sites of the PKD gene.

We have removed all information that may identify this patient to protect the patient’s privacy. The reporting of this case conforms to the CARE guidelines (Gagnier et al., 2013).

## Discussion

We report the clinical manifestations and biological detection consequences of a 15-year-old boy with PKD and CNS-II, who had a novel homozygous pathogenic variant of *PKLR* and a novel heterozygous pathogenic variant of *UGT1A1*. The clinical diagnosis and management of patients with PKD and CNS-II can be challenging due to the complexity of diagnostic evaluations and the wide range of clinical manifestations observed. Fortunately, with the development of sequencing technology, genetic and epigenetic research on complex disorders is becoming more common. The patient was clinically diagnosed with PKD and CNS-II through the next-generation sequencing. It is worth noting that the patient had no neurological symptoms and normal intellectual development up until this point.

PKD is an autosomal-recessive enzyme defect of the glycolytic pathway that causes congenital nonspherocytic hemolytic anemia (Grace and Barcellini, 2020; Kim et al., 2022) caused by bi-allelic pathogenic variants in the *PKLR* gene (Rehman et al., 2022). The detection of decreased PK activity should be first measured for rapid diagnosis. However, we ignored the clinical diagnosis of genetic hemolytic anemia because the hemolytic test was negative and we were not hematology specialists. Crigler-Najjar syndrome is a rare autosomal recessive inherited disorder found in less than 1 per 1.000.000 births. It is characterized by the absence of or decreased activity of UDP-glucuronosyltransferase. The severity of the disease is determined by the level of enzyme production required for the glucuronidation of bilirubin (Tcaciuc et al., 2021). In summary, PKD and Crigler-Najjar syndrome are distinct genetic disorders with different underlying mechanisms and clinical presentations.

The *PKLR* gene is located on chromosome 1q21 and contains 12 exons. With the rapid development of sequencing, genetic, and epigenetic research on complex disorders, multiple mutation sites in PKD have been detected (Canu et al., 2016). The mutation patterns of *PKLR* identified in PKD patients include gene deletion, missense mutation, nonsense mutation, and splicing connection mutation. Among them, missense mutations in gene coding regions are the most common, accounting for approximately 72%, and c.1529G > A is more common in the United States and central Europe, c.1456C>T is more common in southern Europe, and c.1468C>T is more common in Asia (Zanella and Bianchi, 2000; Pissard et al., 2006; Warang et al., 2013). By analyzing the clinical diagnosis of 72 PKD cases, Chinese researchers found that most of the mutation sites were concentrated in the PK barrel domain (67.5%) and the eα/β domain (27.5%), which are both important functional domains of PK (Gao, 2019; Pan and Yao, 2019; Xia et al., 2019). The clinical manifestations of PKD patients are quite different due to the different mechanisms of different site mutations affecting the structure and function of PK. The pathogenicity rating is based on the ACMG and AMP criteria for genetic variation, the c.1276C>T (p.Arg426Trp) is reported for the first time in this paper, and various prediction results suggest that the variant has a high probability of causing deleterious effects on gene and protein structure or function, which has certain academic reference significance. Approximately 130 kinds of *UGT1A1* gene mutations have been found so far, and there are great differences between different regions, different races, and different individuals. Unlike gene mutations of PKD, common mutations in the *UGT1A1* gene in patients with CNS-I include changes in intron splicing donor and acceptor sites, exon skipping, deletion mutations, missense mutations, insertion mutations, or stop codon formation, etc., resulting in a complete lack of UGT, which can lead to severe hyperbilirubinemia and often death in the early neonatal period due to irreversible brain damage caused by kernicterus. However, CNS-II patients mainly have point mutations in the *UGT1A1* gene, especially single-base substitution missense mutations, and the common mutation site is exon 1 missense mutation. A rare heterozygous pathogenic variant of c.-55_-54insAT, c.1091C > T (p.Pro364Leu) in *UGT1A1* is reported in this paper. The former belongs to the upstream mutation of the heterozygous gene, while the latter belongs to the heterozygous missense variation. However, research has shown that CNS can lead to clinical disease only in the case of homozygous mutation and compound heterozygous variants (Takeuchi et al., 2004; Hsieh et al., 2007). Therefore, according to comprehensive judgment, pathological and genetic mutation changes are based on the patient’s clinical characteristics; the patient carried a pathogenic mutation of *PKLR,* which was the main cause of hyperbilirubinemia. CNS-II may be involved in the pathophysiological process in this patient. At present, there is no clear treatment for PKD and CNS-II. The current treatment strategy involves the avoidance of triggers and the use of corticosteroids, regular blood transfusion to maintain hemoglobin levels, and splenectomy performed when necessary (Grace et al., 2018).

## Conclusion

In this study, we report the unique clinical course of a patient with PKD and CNS-II. Genetic testing helped to rapidly identify a potential association, with a novel homozygous pathogenic variant of *PKLR* and a novel heterozygous variant of *UGT1A1*, which expands the phenotypic and molecular spectrum of *PKLR* and *UGT1A1* gene disorders and also emphasizes the importance of combining both targeted next-generation sequencing and detailed clinical evaluation. The identification of these novel variants highlights the complexity of these genetic disorders and emphasizes the importance of comprehensive genetic analysis in diagnosing and managing patients. By combining advanced sequencing technologies with detailed clinical evaluations, we can uncover unique genetic profiles and gain insights into the underlying mechanisms of disease.

## Data Availability

The datasets for this article are not publicly available due to concerns regarding participant/patient anonymity. Requests to access the datasets should be directed to the corresponding author.

## References

[B1] Bhandari J. Thada P. K. Yadav D. (2023). “Crigler Najjar syndrome,” in StatPearls. (Treasure Island (FL)). 32965842

[B2] Canu G. De Bonis M. Minucci A. Capoluongo E. (2016). Red blood cell PK deficiency: An update of PK-LR gene mutation database. Blood Cells Mol. Dis. 57, 100–109. 10.1016/j.bcmd.2015.12.009 26832193

[B3] Gagnier J. J. Kienle G. Altman D. G. Moher D. Sox H. Riley D. (2013). The CARE guidelines: Consensus-based clinical case reporting guideline development. Headache 53, 38–43. 10.7453/gahmj.2013.008 PMC3833570 24416692

[B4] Gao J. (2019). PKLR gene mutation in neonatal pyruvate kinase deficiency: A case report and literature review. Shijiazhuang, China: Master, Hebei Medical University.

[B5] Grace R. F. Barcellini W. (2020). Management of pyruvate kinase deficiency in children and adults. Blood 136, 1241–1249. 10.1182/blood.2019000945 32702739

[B6] Grace R. F. Bianchi P. Van Beers E. J. Eber S. W. Glader B. Yaish H. M. (2018). Clinical spectrum of pyruvate kinase deficiency: Data from the pyruvate kinase deficiency Natural history study. Blood 131, 2183–2192. 10.1182/blood-2017-10-810796 29549173

[B7] Hsieh T. Y. Shiu T. Y. Huang S. M. Lin H. H. Lee T. C. Chen P. J. (2007). Molecular pathogenesis of Gilbert's syndrome: Decreased TATA-binding protein binding affinity of UGT1A1 gene promoter. Pharmacogenet Genomics 17, 229–236. 10.1097/FPC.0b013e328012d0da 17496722

[B8] Kim M. Lee S. Y. Kim N. Lee J. Kim D. S. Park J. (2022). Case report: Compound heterozygosity in PKLR gene with a large exon deletion and a novel rare p.Gly536Asp variant as a cause of severe pyruvate kinase deficiency. Front. Pediatr. 10, 1022980. 10.3389/fped.2022.1022980 36533240 PMC9752143

[B9] Pan Y. Yao H. (2019). A family report of erythrocyte pyruvate kinase deficiency. Chin. J. Hematol. 40, 749. 10.3760/cma.j.issn.0253-2727.2019.09.007 PMC7342443 31648476

[B10] Pissard S. Max-Audit I. Skopinski L. Vasson A. Vivien P. Bimet C. (2006). Pyruvate kinase deficiency in France: A 3-year study reveals 27 new mutations. Br. J. Haematol. 133, 683–689. 10.1111/j.1365-2141.2006.06076.x 16704447

[B11] Rehman A. U. Rashid A. Hussain Z. Shah K. (2022). A novel homozygous missense variant p.D339N in the PKLR gene correlates with pyruvate kinase deficiency in a Pakistani family: A case report. J. Med. Case Rep. 16, 66. 10.1186/s13256-022-03292-z 35168679 PMC8848962

[B12] Richards S. Aziz N. Bale S. Bick D. Das S. Gastier-Foster J. (2015). Standards and guidelines for the interpretation of sequence variants: A joint consensus recommendation of the American College of medical genetics and Genomics and the association for molecular Pathology. Genet. Med. 17, 405–424. 10.1038/gim.2015.30 25741868 PMC4544753

[B13] Takeuchi K. Kobayashi Y. Tamaki S. Ishihara T. Maruo Y. Araki J. (2004). Genetic polymorphisms of bilirubin uridine diphosphate-glucuronosyltransferase gene in Japanese patients with Crigler-Najjar syndrome or Gilbert's syndrome as well as in healthy Japanese subjects. J. Gastroenterol. Hepatol. 19, 1023–1028. 10.1111/j.1440-1746.2004.03370.x 15304120

[B14] Tcaciuc E. Podurean M. Tcaciuc A. (2021). Management of Crigler-Najjar syndrome. Med. Pharm. Rep. 94, S64–S67. 10.15386/mpr-2234 34527915 PMC8411811

[B15] Warang P. Kedar P. Ghosh K. Colah R. (2013). Molecular and clinical heterogeneity in pyruvate kinase deficiency in India. Blood Cells Mol. Dis. 51, 133–137. 10.1016/j.bcmd.2013.05.006 23770304

[B16] Xia Y. Liang Q. Shi H. Gao Y. Xu Q. Jin M. (2019). PKLR gene novel heterozygous mutation in children with pyruvate kinase deficiency. Chin. J. Obstetrics Gynecol. Pediatr. Electron. Ed. 15, 547–553. 10.3877/cma.j.issn.1673-5250.2019.05.011

[B17] Zanella A. Bianchi P. (2000). Red cell pyruvate kinase deficiency: From genetics to clinical manifestations. Baillieres Best. Pract. Res. Clin. Haematol. 13, 57–81. 10.1053/beha.1999.0057 10916678

