# Supplementary material for: Case report: A rare case of pyruvate kinase deficiency and Crigler-Najjar syndrome type II with a novel pathogenic variant of PKLR and UGT1A1 mutation
Source: Front Genet. 2023 Aug 21;14:1229271. doi: 10.3389/fgene.2023.1229271 (PMC10475990; doi:10.3389/fgene.2023.1229271)
Supplement: Supplementary file 1 [file Table1.pdf]

Table S1. The pathological parmeter of patient with Pyruvate Kinase Deficiency on admission

Method	Index	Result
Immunohistochemistry	HBsAg	(-)
	HBcAg	(-)
	Hepatocyte	(+)
	CD10 (bile capillary)	(+)
	CD3 (hepatic lobules)	(+)
	CD20 (few inflammatory cells)	(+)
	CD34 (blood vessel and partial capillary)	(+)
	CD68 (Kupffer cell in the hepatic sinus)	(+)
	CD68 (Macrophages in the portal area)	(+)
	CK7/CK19 (interlobular bile duct)	(-)
	MUM-1 (individual inflammatory cells)	(+)
Special staining	Copper staining	(-)
	Iron staining	(+)
	Masson staining	(-)
	PAS-D (activated macrophages)	(+)
	PAS	(-)
	Reticular fiber dyeing	(-)

Note: WBC: White Blood Cell; RBC: Red Blood Cell; HGB: Hemoglobin; MCV: Mean Corpuscular Volume; MCHC: Mean Corpuscular Hemoglobin Concentration; MCH: Mean Corpuscular Hemoglobin; HCT: Hematocrit; Ret: Reticulocyte; PLT: Platelets; ALT: alanine transaminase; AST: aspartate aminotransferase; TBIL: total bilirubin; DBIL: direct bilirubin; IBIL: indirect bilirubin; ALP: alkaline phosphatase; GGT: gamma-glutamyl transferase; TBA: total bile acid; HBsAg: Hepatitis B surface antigen; HBsAb: Hepatitis B surface antibody; HBeAg: Hepatitis B e Antigen; HBeAb: Hepatitis B e antibody; HBcAb: Hepatitis B c antibody; HBV: Hepatitis B virus; HCV-Ab: hepatitis C virus antibody; HAV: Hepatitis A virus; HDV: Hepatitis D virus; HEV: Hepatitis E virus; CMV: cytomegalovirus; HSV: herpes simplex virus; EBV: Epstein-Barr virus; ANA: antinuclear antibodies; TSH: Thyroid Stimulating Hormone; FT3: free triiodothyronine 3; FT4: free triiodothyronine 4; PNH: Paroxysmal nocturnal hemoglobinuria; G-6-PD: Glucose-6-phosphate Dehydrogenase; IgG: immunoglobulin G; IgA: immunoglobulin A; IgM: immunoglobulin M; AFP: alpha fetoprotein; CEA: carcinoembryonic antigen; PAS: Periodic acid-schiff stain.
